# Vitamin D Signaling in Neurodegenerative Disorders: Mechanisms, Therapeutic Potential, and Clinical Implications

**DOI:** 10.3390/nu18132082

**Published:** 2026-06-25

**Authors:** Naveen Soni, Nabendu Debnath, Ella Rekapally, Ayaan Jabbar, Suresh C. Tyagi, Bhawana Bissa, Neetu Tyagi

**Affiliations:** 1 Department of Biochemistry, Central University of Rajasthan, Ajmer 305817, Rajasthan, India; 2 Centre for Molecular Biology, Central University of Jammu, Rahya-Suchani, Bagla, Samba 181143, Jammu & Kashmir, India; 3 Department of Physiology, School of Medicine, Health Sciences Centre, University of Louisville, A-1201, Louisville, KY 40202, USA

**Keywords:** vitamin D, neurodegenerative diseases, neuroinflammation, oxidative stress, blood–brain barrier, Alzheimer’s disease, mitochondrial dysfunction

## Abstract

Vitamin D has long been recognized for its role in calcium homeostasis and bone metabolism; however, it is now emerging as an important regulator of central nervous system (CNS) function. Recent evidence suggests that vitamin D signaling contributes to the pathogenesis and progression of several neurodegenerative disorders. Vitamin D exerts neuroprotective effects through multiple mechanisms, including regulation of calcium homeostasis, modulation of immune responses, reduction in oxidative stress, stimulation of neurotrophic factors, and maintenance of blood–brain barrier (BBB) integrity. Vitamin D receptors and metabolizing enzymes are widely distributed across several brain regions, highlighting their direct involvement in neuronal function. This review summarizes the biosynthesis, metabolism, and signaling pathways of vitamin D. It explores its role in neurodegenerative diseases such as Alzheimer’s disease (AD), Parkinson’s disease (PD), multiple sclerosis (MS), amyotrophic lateral sclerosis (ALS), stroke, and traumatic brain injury (TBI). Evidence from experimental and clinical studies indicates that vitamin D deficiency is associated with an increased risk and severity of these conditions, while supplementation may provide therapeutic benefits.

## 1. Introduction

Neurodegenerative disorders are driven by multifactorial processes such as neuroinflammation, oxidative stress, and protein aggregation. Emerging evidence highlights vitamin D as a neuroactive secosteroid hormone with roles extending beyond calcium homeostasis to include regulation of neuronal function and immune responses in the central nervous system (CNS). There are two forms of vitamin D in the body: vitamin D_2_ and vitamin D_3_. Vitamin D_3_ is formed in the skin from a cholesterol derivative by the action of UV radiation from the sun [1]. The human body produces 90% of its vitamin D, which depends on sunlight exposure. Natural sources of vitamin D_3_ are eggs, cod liver oil, fish oil, and vitamin D_3_-fortified milk [2]. Vitamin D_2_ is primarily obtained from plant-based and fungal sources, particularly UV-exposed mushrooms and fortified foods. Additionally, at the beginning of the metabolic activity of vitamin D in the liver, it is hydroxylated at carbon 25, which represents the initial step in the metabolic activation pathway of vitamin D [3].

Vitamin D is synthesized in the skin when 7-dehydrocholesterol absorbs UVB radiation, forming pre-vitamin D_3_, which later isomerizes to vitamin D_3_. Vitamin D is subsequently metabolized in the liver to 25-hydroxyvitamin D (25(OH)D) and in the kidneys to the biologically active form 1,25-dihydroxyvitamin D_3_ (calcitriol), which regulates calcium and phosphate homeostasis and supports skeletal health. Because vitamin D receptors are present in many tissues, vitamin D signaling influences numerous biological pathways, and its deficiency has been associated with several chronic diseases.

After entering target tissues, vitamin D and its metabolites bind to the vitamin D receptor (VDR), a nuclear receptor transcription factor in the cytosol [4]. Moreover, VDR was discovered in the cerebellum, thalamus, hypothalamus, posterior ganglia, hippocampus, olfactory system, and temporal and orbital brain regions. It has been emphasized that the etiopathogenesis of various neurodegenerative diseases, such as multiple sclerosis (MS), Parkinson’s disease (PD), Alzheimer’s disease (AD), and amyotrophic lateral sclerosis (ALS), could be affected by environmental and genetic factors related to vitamin D and VDR concentration [5]. Key vitamin D-mediated signaling pathways and their functional consequences in the CNS are summarized in Table 1. Calcitriol has been shown to exert a strong regulatory effect on nerve growth factor (NGF)-mediated signal transduction, as demonstrated in neuronal cells. Consequently, it may play a crucial role in the formation of brain neurons [6]. Many neurodegenerative diseases have overlapping pathologies, such as inflammation, protein aggregation, oxidative damage, and demyelination. The role of vitamin D in the nervous system has gained more interest in recent years. There are many vitamin D-related enzymes and receptors in the brain, and vitamin D metabolites can bind to glial and neuronal cells, exerting diverse effects [7]. It has been shown that vitamin D deficiency can exacerbate neuropathological changes, and supplementation can improve outcomes. This review discusses the link between vitamin D and various neurodegenerative diseases, including the mechanisms by which vitamin D contributes to their pathogenesis and its application in their management.

However, the relationship between vitamin D status and biological effects may not be strictly linear, and responses may depend on baseline serum concentration. Vitamin D values are also correlated with threshold effects, which suggests a biological ceiling, in which VDR-mediated neuroprotection becomes less responsive once 25-hydroxyvitamin D levels cross 30–50 ng/mL. Beyond this value, various counter-regulatory mechanisms are activated, such as the upregulation of the CYP24A1 enzyme [8,9]. A previous study has demonstrated a non-linear association of the serum vitamin D levels with liver cirrhosis and hepatocellular carcinoma (HCC) [10]. The risks and disease-induced mortality have been linked with a U-shaped association curve. Studies have shown that for all causes of mortality, the risk is primarily elevated at lower vitamin D levels (<30 ng/mL), while higher levels (>70 ng/mL) do not appear to significantly increase mortality risk [10]. These findings suggest that the biological effects of vitamin D may depend not only on deficiency status but also on the concentration range, highlighting the importance of considering threshold effects when evaluating its role in neurological diseases.

Based on previous research, many neurological conditions, including PD, AD, and MS, have been associated with vitamin D deficiency [5]. The immunomodulatory and anti-inflammatory properties of vitamin D are attributed to its neuroprotective properties. Vitamin D may help improve symptoms and potentially slow disease progression by reducing oxidative stress and neuroinflammation, stimulating the production of neurotrophic factors, modulating neurotransmitter production, and protecting the blood–brain barrier (BBB). In older individuals, vitamin D deficiency can be prevented and treated to improve depression and cognitive impairment [11]. Collectively, these findings highlight the multifaceted role of vitamin D in neurodegenerative disorders and support the growing interest in its mechanistic and therapeutic relevance.

**Table 1:** Vitamin D-mediated signaling pathways and their functional consequences in the CNS.

Signaling Pathway	Mechanism of Action	Functional Consequences in the CNS	Research Model	References
Genomic vitamin D receptor (VDR) signaling	1,25(OH)_2_D_3_ activates VDR– retinoid X receptor (RXR) complex to regulate gene transcription via VDREs.	Regulates genes involved in neuronal differentiation, synaptic function, and neurotrophic signaling.	Fish	[8,12]
NF-κB signaling pathway modulation	Suppression of NF-κB signaling through IκB stabilization and inhibition of p65 translocation.	Attenuation of neuroinflammation and reduced microglial and astrocyte activation.	Mouse	[13]
Nrf2-mediated antioxidant signaling	Vitamin D-mediated Nrf2 activation with ARE-dependent expression of cytoprotective genes.	Enhanced antioxidant defense with reduced neuronal ROS accumulation.	Mice and the HT22 cell line	[14,15]
MAPK signaling pathway regulation	Vitamin D-mediated modulation of MAPK signaling (ERK, JNK, p38), regulating cell survival and stress responses	Vitamin D-mediated ERK activation with modulation of JNK/p38 apoptosis-associated signaling.	Rat	[16,17]
Wnt/β-catenin signaling pathway interaction	Vitamin D-mediated modulation of Wnt/β-catenin signaling via regulation of β-catenin activity.	This promotes neuronal development, synaptic plasticity, and maintenance of neural circuits.	Mouse	[18]
PI3K/Akt signaling pathway activation	Vitamin D-mediated PI3K/Akt activation regulates survival and apoptosis signaling	Enhanced neuronal survival, metabolic homeostasis, and cellular stress resistance	Mice and Rat	[19,20]
Calcium signaling regulation	Vitamin D-mediated regulation of calcium homeostasis (calbindin, L-type Ca^2+^ channels).	Prevention of Ca^2+^ overload and excitotoxicity, maintaining neuronal function	-	[21,22]
Toll-like receptor (TLR) signaling modulation	Vitamin D-mediated modulation of TLR2/4–MyD88 innate immune signaling.	This leads to controlled activation of microglia and reduced production of pro-inflammatory mediators.	Rat	[23,24]

## 2. Vitamin D Biosynthesis, Signaling, and Regulation

**Figure 1:**
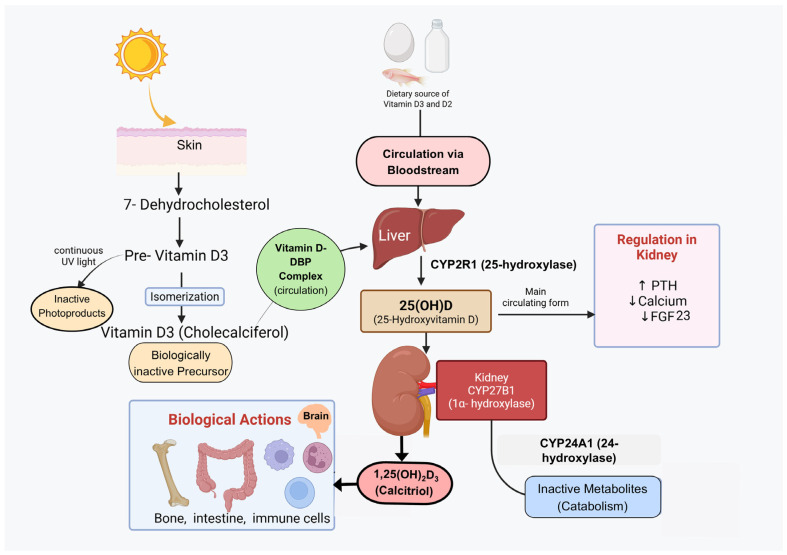
The biosynthesis, regulation and metabolic pathway of vitamin D: Vitamin D is produced in the skin by UV exposure or obtained from diet and transported in the blood. It is converted in the liver to 25(OH)D and further activated in the kidney to 1,25(OH)_2_D_3_ (calcitriol). FGF23, PTH, and calcium regulate this process. Active vitamin D exerts biological effects on bone, intestine, immune cells, and the brain, supporting calcium homeostasis, immune regulation, and overall physiological functions.

Vitamin D is a fat-soluble nutrient, synthesized when 7-dehydrocholesterol is exposed to UV light, the B ring is broken, and it forms pre-D_3_ [25]. Pre-D_3_ isomerizes to D_3_, although tachysterol and lumisterol are produced when exposed to continuous UV light. D_3_ is linked to DBP and is preferentially extracted from the skin (Figure 1). Whether taken orally or through the skin, vitamin D is converted by the liver and other tissues into its main circulating form, 25(OH)D [26]. The circulating 25-hydroxyvitamin D levels are generally maintained between 20 and 50 ng/mL (with ~30 ng/mL often considered optimal) to ensure an adequate concentration of substrate. It has also been reported that 25-hydroxyvitamin D levels below 20 ng/mL are considered deficient, levels between 20 and 30 ng/mL are considered insufficient, and markedly elevated levels are associated with hypervitaminosis D (vitamin D toxicity) [27]. Consuming high doses of vitamin D (>10,000 IU/day) is linked to hypervitaminosis D, can cause symptoms of hypercalcemia, apathy, and several other linked renal and neurological disorders. The most significant enzyme with 25-hydroxylase activity is CYP2R1. The enzyme CYP27B1 then further metabolizes 25(OH)D to 1,25(OH)_2_D_3_, mostly in the kidney. However, it is also present in other organs, including immune system cells, parathyroid gland cells, and various epithelial cells [1,28]. The primary hormonal form of vitamin D, 1,25(OH)_2_D_3_, is in charge of most of its biological effects. The major vitamin D-mediated signaling pathways and their functional roles in the CNS are summarized in Table 1. The kidney’s generation of 1,25(OH)_2_D_3_ is strictly regulated; parathyroid hormone (PTH) stimulates it, while calcium, phosphate, and FGF23 decrease it (Figure 1) [29]. 1,25(OH)_2_D_3_ lowers its own cellular levels primarily by promoting its catabolism via induction of CYP24A1, the 24-hydroxylase. The extrarenal production of 1,25(OH)_2_D_3_, as in keratinocytes and macrophages, is regulated differently and is mainly stimulated by cytokines such as tumor necrosis factor alpha (TNFα) and interferon gamma (IFNγ) [30]. The CYP24A1 enzyme hydroxylates 25(OH)D and 1,25(OH)_2_D_3_ at position 24 to produce 24, 25(OH)_2_D and 1, 24, 25(OH)_3_D, respectively. Although 24, 25(OH)_2_D and 1, 24, 25(OH)_3_D have their own biologic functions, this 24-hydroxylation is typically the initial step in the catabolism of these active metabolites to the end product of calcitroic acid [31]. Additionally, CYP24A1 exhibits 23-hydroxylase activity, which produces an alternative product. The ratio of 23-hydroxylase to 24-hydroxylase activity in CYP24A1 varies among species, though in humans, 24-hydroxylase activity is more prevalent. CYP24A1 is expressed as widely as CYP27B1 [32]. In most tissues, 1,25(OH)_2_D_3_ induces CYP24A1, a crucial feedback mechanism that prevents vitamin D toxicity. In the kidney, FGF23, calcium, and phosphate activate CYP24A1, while PTH inhibits it. This is the exact reverse of how these hormones and minerals affect CYP27B1. Other tissues, however, do not exhibit this control. Since CYP24A1 is either absent or malfunctioning in macrophages, hypercalcemia and hypercalciuria resulting from elevated 1,25(OH)_2_D_3_ can happen in conditions such as granulomatous disorders like sarcoidosis, where macrophage production of 1,25(OH)_2_D_3_ is enhanced without CYP24A1 counter-regulation [1,33]. Together, these tightly regulated biosynthetic and catabolic steps maintain vitamin D homeostasis and prevent both deficiency-related dysfunction and toxicity, underscoring the importance of context-dependent vitamin D signaling in CNS physiology. Beyond its biosynthesis and regulatory control, the clinical relevance of vitamin D may also depend on the disease stage. Available evidence suggests that vitamin D may be more effective in preclinical or early symptomatic phases, particularly when deficiency is corrected. In contrast, its impact in advanced disease appears less consistent and may be limited to supportive or symptomatic benefits. The temporal pattern of vitamin D in neurodegenerative disorders seems most crucial during the pre-clinical and early symptomatic stages [34,35]. Research suggests that vitamin D mainly acts as an initial modulator, controlling calcium levels and reactive oxygen species (ROS), and reducing neuroinflammation before irreversible synaptic damage happens [36]. In AD, macrophages clear amyloid-β (Aβ) most effectively during early accumulation. As the disease advances into neurodegeneration, the effectiveness of vitamin D supplementation diminishes due to the influence of the VDR [37]. Therefore, vitamin D plays a crucial role in preventing disease and in early intervention. However, its impact during late stages is probably limited to symptom management rather than altering disease progression.

### 2.1. Neuroprotective Mechanism of Vitamin D

Vitamin D plays a multifaceted neuroprotective role in the CNS by regulating calcium homeostasis, modulating inflammatory responses, exerting antioxidant effects, supporting neurotrophic factors and neurogenesis, influencing neurotransmission and maintaining BBB integrity.

In terms of calcium homeostasis, vitamin D, particularly its active form 1,25-dihydroxycholecalciferol, modulates intracellular calcium levels in neurons by regulating calcium-binding proteins and calcium channels. This regulation helps prevent excitotoxicity caused by excessive calcium influx, a common pathway leading to neuronal injury and death, thus maintaining neuronal health [38]. Dysregulation of calcium homeostasis is implicated in neurodegeneration, including AD, where perturbed intracellular calcium contributes to synaptic dysfunction and neuronal apoptosis [39].

Vitamin D also exerts anti-inflammatory effects by suppressing pro-inflammatory cytokines such as TNF-α and interleukin-6 (IL-6) while promoting anti-inflammatory cytokines like IL-10. This immunomodulatory role reduces neuroinflammation, which is a key contributor to chronic neurodegenerative processes including MS, AD, and PD [40,41]. It acts in glial cells, including astrocytes and microglia, to regulate immune function and mitigate inflammatory damage [42].

Regarding antioxidant properties, vitamin D enhances the expression of antioxidant enzymes such as glutathione peroxidase (GPx) and superoxide dismutase (SOD), which reduce oxidative stress and reactive oxygen species (ROS) accumulation. By modulating oxidative pathways, vitamin D protects neurons from oxidative damage, a critical factor in neuronal degeneration and cognitive decline [43,44].

Vitamin D supports neurotrophic factors, stimulating the synthesis of NGF and brain-derived neurotrophic factor (BDNF), thereby promoting neuronal survival, differentiation, synaptic plasticity, and repair processes. This neurotrophic support aids neural regeneration and functional recovery after injury [45]. It also promotes neural stem cell proliferation and differentiation, contributing to remyelination and neuroprotection in diseases like multiple sclerosis [46].

Modulation of neurotransmission by vitamin D involves regulating the synthesis and release of key neurotransmitters such as dopamine, serotonin, and acetylcholine. These neurotransmitters are vital for cognitive functions and mood regulation, linking vitamin D to psychiatric and neurodegenerative disease prevention [47].

In neurodegenerative disease pathology, vitamin D enhances the clearance of amyloid-beta peptides and reduces tau phosphorylation, mechanisms that mitigate plaque formation and neurofibrillary tangles characteristic of the disease. These actions support the slowing of AD progression through estimated neuroprotective pathways [48]. The association between vascular dysfunction and Alzheimer’s also highlights the role of vitamin D in maintaining BBB integrity, thereby preventing infiltration of neurotoxic substances and immune cells that exacerbate neurodegeneration [49].

Vitamin D supports BBB integrity by regulating tight junction proteins and reducing BBB permeability. This is crucial for maintaining the selective barrier function of the BBB to protect the CNS from harmful agents. Vitamin D decreases leukocyte recruitment into the CNS and counteracts neuroinflammation, promoting neuronal survival and brain homeostasis [50,51]. BBB dysfunction is a known contributor to multiple neurodegenerative disorders; therefore, vitamin D’s role in preserving BBB integrity has therapeutic implications [52].

In summary, vitamin D acts as a critical modulator of multiple neuroprotective mechanisms: it regulates neuronal calcium to prevent excitotoxicity; controls inflammatory and oxidative stress responses to protect neurons; promotes neurotrophic support and neurogenesis; regulates neurotransmitter systems vital for cognition and mood; reduces pathological hallmarks of Alzheimer’s; and maintains BBB integrity to safeguard the brain’s microenvironment. These combined effects underscore the importance of vitamin D in maintaining brain health and preventing or slowing the progression of neurodegenerative diseases [36,53,54,55].

### 2.2. Vitamin D Signaling in Specific Neurodegenerative Diseases

#### 2.2.1. Alzheimer’s Disease

**Figure 2:**
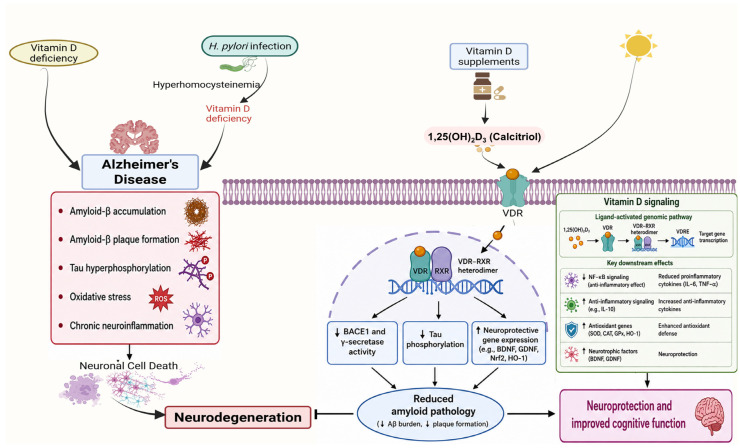
Crosslink between vitamin D deficiency and its regulation in Alzheimer’s Disease: Vitamin D deficiency contributes to Alzheimer’s disease by promoting amyloid-β accumulation, tau hyperphosphorylation, oxidative stress, and chronic neuroinflammation, leading to neuronal cell death and neurodegeneration. The active form of vitamin D, 1,25(OH)_2_D_3_ (calcitriol), binds to the vitamin D receptor (VDR) and regulates gene expression via VDR–RXR signaling. This reduces amyloidogenic processing (BACE1 and γ-secretases), decreases tau phosphorylation, suppresses pro-inflammatory cytokines, and enhances antioxidant and neuroprotective responses. It also helps to reduce amyloid plaque formation, and supports neuroprotection and improves cognitive function.

AD is one of the leading causes of dementia, often characterized by the progressive loss of memory and cognition [56]. Senile plaques are a hallmark of AD and form when Aβ peptides begin to aggregate in the brain [57] (Figure 2). It was anticipated that low vitamin D levels would be linked to a higher risk of AD and compromised cognitive performance. It has been documented that adding vitamin D to Alzheimer’s treatment improves cognitive abilities. The risk of dementia and AD has been found to be considerably increased by vitamin D deficiency. Different clinical studies evaluating this association are summarized in Table 2. According to another study, it was unclear how cognitive function and 25(OH)D_3_ levels were related [58]. Chronic inflammation and vitamin D insufficiency impair cognitive function and have an impact on neurodegeneration in the elderly population. Decreased vitamin D levels and depression have also been found to be strongly correlated [59]. As people age, vitamin D insufficiency becomes increasingly prevalent and is linked to several disorders. Treatment with vitamin D_3_ has a favorable major impact on life expectancy and quality of life. Combined treatment with memantine and vitamin D has been reported to improve cognitive function in patients with AD and may provide therapeutic benefits by slowing disease progression [37]. According to another investigation, the neurogenesis process contributes favorably to the substantial protection against neurological disorders that stem-cell application and vitamin D treatment offer. Low vitamin D levels have a major role in the onset and progression of the majority of neurodegenerative disorders, including AD, since it has been found that vitamin D levels are effective in neurological diseases [45]. As a result, it is stressed that vitamin D receptor agonists are crucial to vitamin D-based research and that maintaining vitamin D levels at a specific level throughout life is crucial. According to reports, vitamin D receptor suppression unavoidably causes aging and neurodegeneration and speeds up the process of neurodegeneration in conjunction with Aβ toxicity [60]. It has been shown that long-term vitamin D deficiency promotes neuron death, which in turn leads to aging and neurodegeneration. In another molecular investigation, it was found that IL-34, induced by 1α,25(OH)_2_D_3_, comprises a key mechanism in the protective effect of vitamin D in AD [61]. It has also been highlighted that *Helicobacter pylori* infection plays a significant role in neurodegenerative diseases like PD and MS, including AD, by influencing vitamin D levels through hyperhomocysteinemia [62]. It has been shown that increasing PGC-1α production in the hippocampal regions of AD mice provides substantial protection by significantly reducing Aβ plaque formation through activation of VDRs [63]. Another in vitro study found that resveratrol and vitamin D together suppressed tau phosphorylation. The beneficial effects of vitamin D application (1000 IU/day) on learning mechanisms have been established. Through its antioxidative, anti-inflammatory, and anti-apoptotic properties, vitamin D also offers significant protection against a variety of neurodegenerative disorders [64]. Additionally, recent research demonstrated that vitamin D and its analogs inhibited the synthesis of Aβ and promoted its breakdown in neuroblastoma cells or in the brains of mice lacking vitamin D. They stated that BACE1 and γ-secretases, which produce Aβ, are impacted by this inhibition (Figure 2). As a result, vitamin D’s impact on AD has been shown, and it has been proposed that it could help prevent the condition [65]. Similarly, it has been highlighted that vitamin D’s preventive impact in AD is significant for other neurodegenerative illnesses as well. Through its regulation of intracellular signaling pathways and calcium homeostasis, vitamin D exerts neuroprotective effects that may attenuate neurodegenerative progression. Vitamin deficiencies have also been associated with impaired cognitive function; therefore, supplementation with vitamin B complex, folic acid, and vitamin D has been suggested to support neurological health. In a similar vein, it has been proposed that vitamin D supplements could benefit people with impaired cognitive function [60]. Gezen-Ak and colleagues’ investigation revealed a strong correlation among vitamin D, VDR, and hippocampal tissue, suggesting that vitamin D deficiency may be linked to AD [66]. Similar findings were also documented, showing that vitamin levels and AD are significantly correlated [67]. The biggest difference between the AD and control groups was observed for vitamin C, but there were also noticeable differences in vitamin D, E, folate, A, and B12. These results imply that vitamin deficiencies are major contributors to AD pathogenesis and could be a target for future treatment approaches [68].

#### 2.2.2. Parkinson’s Disease

**Figure 3:**
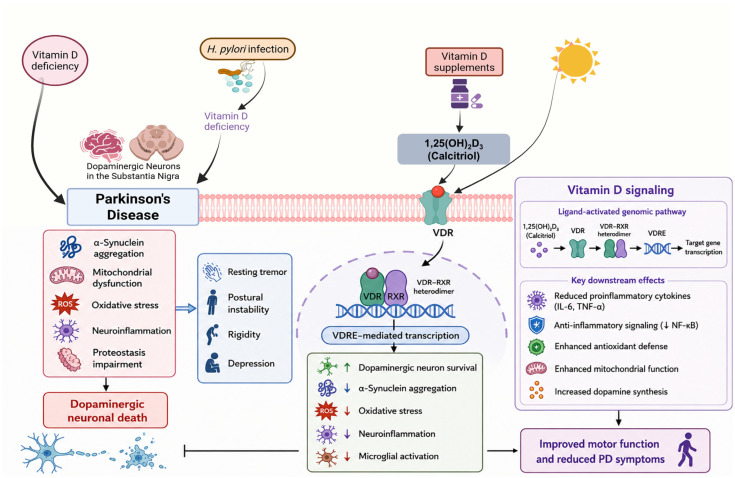
The deficiency of vitamin D and its consequences in the progression of Parkinson’s disease: Vitamin D deficiency contributes to Parkinson’s disease by promoting α-synuclein aggregation, mitochondrial dysfunction, oxidative stress, and neuroinflammation, leading to the loss of dopaminergic neurons and motor dysfunction. The active form of vitamin D, 1,25(OH)_2_D_3_ (calcitriol), activates VDR–RXR-mediated transcription, which reduces inflammation and oxidative stress, improves mitochondrial function, and enhances dopaminergic neuron survival and dopamine production.

PD, a common neurological disorder expected to affect more elderly adults and render them incapable of performing various activities effectively, has been associated with a deficiency of dopamine in the brain due to a failure of nerve cells to generate enough dopamine in this context [86] Common symptoms of PD include tremors, rigidity of the limbs and trunk, impaired balance and coordination, and generalized slowness of movement (bradykinesia) [87]. Additionally, individuals suffering from PD are also likely to experience dysphagia, as well as depression and sleep problems [88]. PD has been identified as a condition in which dopaminergic neurons in a part of the substantia nigra, identified as pars compacta, fail to perform adequately in providing dopamine, a condition indicative of this disease [89] (Figure 3). The mitochondria in PD are vulnerable to oxidative stress and neuroinflammation, and vitamin D can alleviate this disease in various ways by reducing oxidative stress via pathways including NF-kB and Nrf2, and by safeguarding astrocytes from ROS [90].

Chronic neurodegenerative diseases can also be worsened in the absence of adequate levels of vitamin D. The beneficial effect of vitamin D is attributed to its constituent antioxidative defense, calcium homeostasis, immune response, neurotransmission, and detoxification pathways, which might protect against PD [91]. Vitamin D deficiency is thought to lead to the cell death of the substantia nigra’s dopaminergic cells, thereby leading to PD. In a study of PD, researchers observed that patients with PD have significantly lower vitamin D levels [92]. In a study done by Sanchez et al. on rats whose model mimics PD, researchers observed reduced tyrosine hydroxylase staining in the rats’ substantia nigra, whereas BDNF, and GDNF expression increased before treatment [93]. This indicates that 1,25(OH)_2_D_3_, a form of vitamin D, would protect the rats’ dopamine-producing cells from damage. A study done on the Chinese population revealed that no correlation is observable between PD sufferers’ genetic variants of the VDR, and PD [94]. The nigrostriatal system is significantly affected in PD, a disease where vitamin D is especially abundant. The mechanisms by which vitamin D regulates phosphatase homeostasis, thereby protecting against the adverse consequences of excessive L–DOPA, are an essential aspect of vitamin D’s importance alongside its neuronal homeostatic, developmental, and maturation pathways [95]. Additionally, an abnormal form of vitamin D acts at the molecular level; upon activation, it can reduce Klotho levels, thereby impairing dopaminergic neurotransmission [96].

To begin with, there is a demonstrated relationship between neurological disorders and variances/mutations in the vitamin D receptor genetics [97]. Moreover, there is a demonstrated relationship between the development and progression of PD and levels of 25-hydroxyvitamin D_3_ [9]. Vascular endothelial dysfunction and neuroinflammation are commonly observed in PD, and VDRs have been identified in endothelial tissues, suggesting a potential regulatory role of vitamin D in vascular and inflammatory processes. Elevated levels of pro-inflammatory cytokines, including TNF-α and IL-6, have also been shown to contribute significantly to the induction and maintenance of dyskinesia in PD [98].

In other related areas, it is clear that vitamin D may also have a positive effect on matrix metalloproteinases, nerve growth factor, and L-type voltage-gated calcium channels, among other aspects of PD [99]. It therefore becomes apparent that the active form of vitamin D, 1,25(OH)_2_D_3_, binds to specific VDRs within the CNS, thereby contributing to its important neurological functions [100].

Vitamin D_3_, through activation of the VDR, exerts antioxidant and anti-inflammatory effects and may therefore provide neuroprotective benefits in neurodegenerative diseases such as PD. Reduced circulating levels of vitamin D_3_ and calcidiol have been associated with an increased risk of major neurodegenerative disorders, including PD [101]. Vitamin D is a neuroactive steroid with a significant role in the maintenance and possible amelioration of diseases such as ALS, AD, and PD. The Klotho protein in cerebrospinal fluid may be a useful biomarker for aging-related pathways in PD, as its levels are often decreased and correlate with disease severity [102]. In PD, vitamin D deficiency has been reported to be associated with reduced dopamine levels and α-synuclein accumulation. Experimental data demonstrated that vitamin D treatment is neuroprotective [103], particularly by increasing anti-inflammatory cytokines and reducing pro-inflammatory cytokines and supporting clinical evidence is summarized in Table 2. Astrocytes participate in the pathogenesis of PD, contributing to α-synuclein pathology but can protect neurons through mechanisms including α-synuclein clearance. Astrocytes positive for the vitamin D-activating enzyme CYP27B1 have been identified in PD, and those CYP27B1-expressing astrocytes sequester α-synuclein oligomers, showing a possible neuroprotective function. Overall, vitamin D_3_ modulates both neuroinflammation and immune function [104].

#### 2.2.3. Multiple Sclerosis

**Figure 4:**
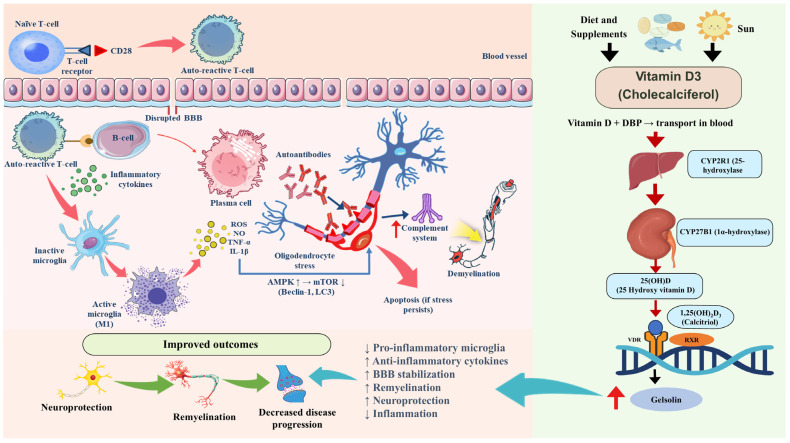
Immunopathogenesis of multiple sclerosis (MS) and immunomodulatory role of vitamin D_3_: Peripheral activation of autoreactive T cells through antigen presentation and co-stimulation (CD28) facilitates their migration across a compromised BBB, resulting in the release of pro-inflammatory cytokines and the activation of microglia and B cells. B cells become plasma cells that make autoantibodies. This activates the complement system and causes immune-mediated demyelination. Inflammatory mediators (ROS, NO, TNF-α, IL-1β) cause oligodendrocyte stress, which disrupts AMPK–mTOR signaling and apoptosis/autophagy pathways, leading to the loss of myelin. Vitamin D_3_ (cholecalciferol), obtained from dietary sources or sunlight, undergoes hydroxylation in the liver (CYP2R1) and kidney (CYP27B1) to produce 1,25(OH)_2_D_3_ (calcitriol), which interacts with the VDR–RXR complex to modulate gene transcription. This signaling stops microglia from being pro-inflammatory, boosts anti-inflammatory cytokines, strengthens the BBB, and helps oligodendrocytes mature and remyelinate.

Impaired immunological response to myelin sheaths is a hallmark of MS, a neurodegenerative illness. Neuronal injury results in the formation of sclerosis plaques surrounding brain neurons. Significant advancements have been made in the therapeutic treatment of MS through the introduction of therapies that impact the peripheral nervous system immunity [105]. MS employs an unidentified substance to initiate a T cell-mediated inflammatory response that demyelinates CNS tissues (Figure 4). Some studies have suggested indirect effects mediated by regulatory T cells. MS patients have reduced regulatory T cells. One powerful immunomodulator is vitamin D [106]. In an experimental animal model of MS, some studies have demonstrated that administering the biologically active hormone 1,25-dihydroxyvitamin D prevents the development and progression of MS [107]. Local paracrine or autocrine effects of vitamin D are predicted by the presence of VDR in neurons, microglia, and the CNS [108]. Serological indicators of disease activity were the subject of a randomized, double-blind, placebo-controlled study. Anti-inflammatory cytokine transforming growth factor-β (TGF-β) and IL-2 levels were significantly reduced after 6 months of 1000 IU cholecalciferol application [109]. For MS patients, vitamin D supplementation is advised, and clinical trial evidence supporting this approach is mentioned in Table 2. According to previous reports, high-dose vitamin D supplementation significantly promotes remyelination and neural repair, particularly in demyelination induced by experimental cuprizone administration, through mechanisms involving oligodendrocyte maturation and astrocyte activation. Therefore, vitamin D supplementation has been recommended for patients with MS [110,111]. It has been proposed that the vitamin D_3_ receptor, which is expressed in oligodendrocytes, could be a novel target for remyelination. Vitamin D-binding protein (DBP) and extracellular gelsolin (GSN) levels were shown to be low in another study, and it was proposed that raising GSN levels could be a novel therapeutic strategy. Inflammatory demyelination and neurodegeneration are the two most significant complications of MS [112]. Thus, vitamin D’s protective effects and its role in immune system regulation are important therapeutic components. The most noteworthy discovery regarding MS is that vitamin D_3_ stimulates the growth of stem cells and promotes neural stem cells to differentiate into oligodendrocytes, a crucial process for remyelination [113]. Consuming more vitamin D is associated with a decreased incidence of MS. Numerous factors, such as vitamin D deficiency, infections, and hormonal imbalance, contribute to the development of inflammation and neurodegeneration in multiple sclerosis [114]. Because of this, vitamin D insufficiency has been proposed as a key risk factor for MS. 1,25(OH)_2_D_3_ offered substantial protection by lowering neuronal loss, inflammation, and demyelination (Figure 4). It has been highlighted that this protection is mostly due to increased Beclin1 expression, a rise in the Bcl-2/Bax ratio, and a decrease in LC3 accumulation, all of which are critical components of apoptosis [54,115].

#### 2.2.4. Amyotrophic Lateral Sclerosis

Progressive motor neuron degeneration in ALS is accompanied by microglial activation, astrogliosis, lymphocyte infiltration, and dendritic cell activation. Additionally, ALS is a progressive motor neuron disease characterized by degeneration of upper and lower motor neurons. ALS exists within a broader motor neuron disease spectrum that includes progressive muscular atrophy (PMA) and primary lateral sclerosis (PLS), and it shares pathological and genetic overlap with frontotemporal dementia (FTD) [116]. ALS-like neurological manifestations have occasionally been reported in hypoparathyroidism, a potentially treatable endocrinopathy. Furthermore, vitamin D administration has been shown to promote axonal regeneration in experimental rat models of ALS [117]. It has been reported that in the experimental rat model of ALS, vitamin D treatment had no effect on the development of leg palsy or the age at which the disease first manifested [117]. For comparison, D_3_ has been shown to have a neuroprotective effect and reduce inflammation in experimental models of TBI [118]. Vitamin D’s neuroprotective effect can also be explained by its ability to lower ROS levels. Vitamin D supplementation shields neurons from apoptosis and cytotoxicity. 

**Figure 5:**
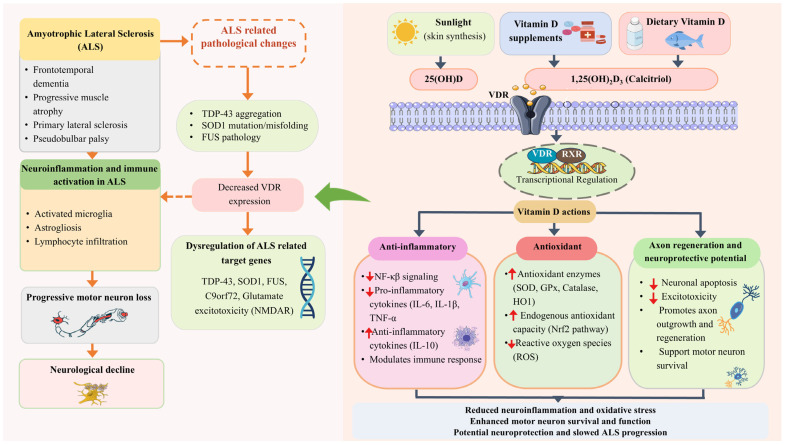
Pathobiology of amyotrophic lateral sclerosis (ALS) and the potential neuroprotective role of vitamin D: ALS is a progressive neurodegenerative disorder characterized by the degeneration of motor neurons, accompanied by microglial activation, astrocytosis, lymphocyte infiltration, and the pathological aggregation of proteins such as TDP-43, SOD1, and FUS. These changes lead to VDR downregulation, excitotoxicity, oxidative stress, inflammation, and progressive weakness. Vitamin D, which comes from sunlight, food, or supplements and is turned into calcitriol, works through the VDR–RXR pathway to control transcription, stop inflammatory signaling, lower ROS, boost antioxidant defenses, and support neuronal survival and axonal regeneration.

Similarly in AD models, according to reports, Aβ significantly reduces VDR expression, which causes neurodegeneration [119] (Figure 5). Many target genes are impacted by Aβ1–42. These have a complex effect on the regulation of the amyloidogenic process, specifically leading to a general overexpression of genes associated with the tau pathway and a general downregulation of NMDAR, ApoE, TREM2, and 1αOHase genes [120,121]. Altered miRNA expression has been implicated in AD, where several miRNAs regulate amyloid precursor protein (APP) processing and Aβ formation, thereby contributing to Aβ pathogenesis. Several miRNAs directly target the 3′UTR or 5′UTR of APP mRNA and regulate BACE1 expression, thereby influencing Aβ generation and neurodegenerative progression in AD [122]. The vitamin D binding protein complex (VitD-dgVDBP) is deglycosylated by 25-OH vitamin D. Likewise, in AD patients, curcumin restores Aβ phagocytosis by peripheral blood mononuclear cells and Aβ clearance through the activation of important genes such as Toll-like receptors (TLRs), VDR, and MGAT3 [123]. Increased neurological issues in ALS are caused by inadequate intake of vitamin E, riboflavin, pyridoxine, folate, cobalamin, Ca, Zn, Mg, and vitamin D. In diseases like ALS, cognitive deficits are a serious issue. Vitamin D has been identified as a key moderating component in the treatment of cognitive impairment in recent years [124]. Vitamin D deficiency has been shown to have detrimental impacts on quality of life, while vitamin D supplementation has demonstrated beneficial effects on cognitive function in patients with ALS. Additionally, because ALS patients have trabecular bone loss, a multidisciplinary approach should be used in their treatment. Vitamin E and vitamin D offer protection against ALS, while dietary components circulating in the blood pose a risk for the disease [125]. Other substances, including linoleic acid, are also advised to reduce the risk of ALS. It should be noted that the choice of vitamins should be tailored to the individual, even though the delaying effect of the ketogenic diet and vitamin supplementation on motor neuron degeneration and the progression of the disease in ALS has been established. Supplementation has been proposed to prevent vitamin D deficiency-related medical consequences in people with ALS and other neurodegenerative disease groups that exhibit limited mobility. Additionally, some research claims that taking vitamins orally has no beneficial effects on the disease’s prognosis [126,127].

#### 2.2.5. Stroke

Stroke remains a leading cause of death and long-term disability, driven by acute ischemic injury and secondary neurovascular dysfunction, including oxidative stress, BBB disruption, neuroinflammation, and neuronal apoptosis. Beyond its classical roles in calcium homeostasis and bone metabolism, vitamin D signaling has emerged as a key regulator of neurovascular health, owing to the widespread expression of the VDR and the vitamin D-activating enzyme 1α-hydroxylase in neurons, glia, endothelial cells, and immune cells within the brain [97,128].

**Figure 6:**
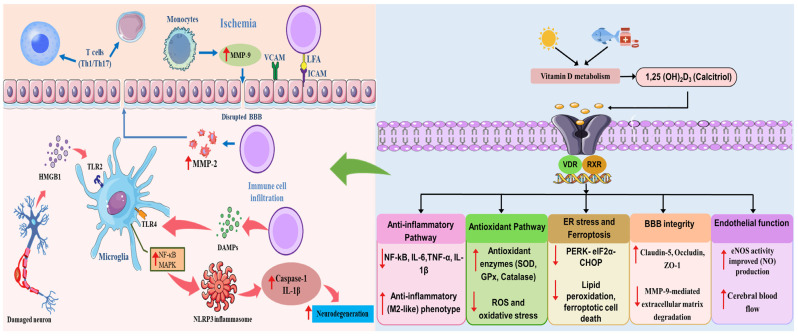
Ischemic stroke pathophysiology and neurovascular modulation by vitamin D signaling: Ischemic injury causes the blood–brain barrier (BBB) to break down by increasing the levels of adhesion molecules (VCAM-1, ICAM-1) and matrix metalloproteinases (MMP-2/9). This lets Th1/Th17 cells and monocytes into the CNS. Damage-associated molecular patterns (DAMPs) released from injured neurons activate microglia through TLR2/4, initiating NF-κB/MAPK signaling, NLRP3 inflammasome activation, and caspase-1-mediated IL-1β release, consequently exacerbating neuroinflammation and neurodegeneration. Vitamin D3, which is turned into calcitriol, works through the VDR–RXR complex to control transcriptional programs that lessen post-ischemic injury. This includes stopping pro-inflammatory signaling, boosting antioxidant defenses, and changing the pathways for ER stress and ferroptosis. Vitamin D also protects the BBB by keeping tight junction proteins (claudin-5, occludin, and ZO-1) in place and lowering MMP-9 activity. It also improves endothelial function by boosting eNOS activity and nitric oxide production.

Preclinical models of cerebral ischemia demonstrate that active vitamin D (1,25-dihydroxyvitamin D_3_) attenuates infarct size and improves functional recovery through modulation of inflammatory and oxidative pathways. Specifically, vitamin D suppresses pro-inflammatory NF-κB signaling and decreases the levels of TNF-α, IL-1β, and IL-6 while promoting anti-inflammatory mediators and shifting microglia toward a reparative phenotype [129,130] (Figure 6). Vitamin D also enhances endogenous antioxidant defenses by increasing SOD and GPx activity, thereby mitigating ROS-mediated neuronal damage after ischemia–reperfusion injury [43]. Recent mechanistic studies further indicate that vitamin D modulates endoplasmic reticulum (ER) stress and ferroptosis pathways, suggesting additional routes by which it may protect ischemic neurons and support cellular homeostasis post-stroke [131]. A critical aspect of post-stroke pathophysiology is BBB breakdown. Vitamin D supports vascular integrity by preserving key tight junction proteins such as claudin-5 and occludin and by attenuating matrix metalloproteinase (MMP-9) activity—processes known to exacerbate BBB permeability in ischemic stroke [50,132]. Moreover, vitamin D enhances endothelial nitric oxide synthase (eNOS) activity and cerebrovascular reactivity, promoting vascular repair and improved blood flow in the post-ischemic brain [133] (Figure 6). Clinically, vitamin D deficiency is highly prevalent among stroke patients and has been linked with greater ischemic stroke severity and poorer short- and long-term outcomes. Prospective observational data reveal that lower serum 25-hydroxyvitamin D levels correlate with increased lesion severity, worse neurological deficits, and poorer functional recovery based on NIH Stroke Scale and modified Rankin Scale scores [134]. Meta-analyses of observational studies corroborate that low vitamin D status is associated with a significantly elevated risk of ischemic stroke as well as poorer post-stroke prognoses, highlighting its potential role as a prognostic biomarker [135,136]. These findings are further summarized in Table 2. Interventional studies suggest that vitamin D supplementation may improve post-stroke outcomes in select populations, particularly among those who are deficient at baseline. Recent systematic reviews and meta-analyses of randomized and controlled studies show that vitamin D supplementation can lead to meaningful improvements in motor function (e.g., Brunnstrom Recovery Stage), NIHSS scores, and modified Rankin Scale outcomes in rehabilitating ischemic stroke patients [78,137]. However, other trials demonstrate mixed results in functional rehabilitation metrics, indicating heterogeneity in response that may depend on dosing strategies, timing, and patient characteristics [138]. Large, well-powered randomized controlled trials are still needed to define optimal supplementation parameters, including dose, duration, and identification of responder subgroups [139]. Taken together, vitamin D signaling appears to modulate key neurovascular processes after ischemic stroke, including inflammation, oxidative stress, ER stress/ferroptosis, and BBB integrity. While observational evidence consistently links vitamin D deficiency to worse stroke incidence and outcomes, the translational impact of supplementation remains an active area of research. Nonetheless, current evidence supports the clinical rationale for screening and correcting vitamin D deficiency as a potentially beneficial adjunct to stroke prevention and recovery strategies [99].

## 3. Conclusions

Vitamin D signaling plays an important role in maintaining CNS function and protecting against neurodegenerative processes. Through its active form, calcitriol, vitamin D regulates several biological mechanisms such as calcium homeostasis, immune modulation, antioxidant defense, neurotrophic support, and maintenance of BBB integrity. These mechanisms contribute to neuron survival and may help slow the progression of neurological disorders. Increasing evidence suggests that vitamin D deficiency is associated with several neurodegenerative diseases, including AD, PD, MS, ALS and ischemic stroke. Some studies indicate that vitamin D supplementation may provide neuroprotective benefits and improve disease outcomes in certain populations. Recent clinical evidence indicates that while vitamin D exhibits robust mechanistic neuroprotective properties, further large-scale clinical studies are required to better understand the precise mechanisms of vitamin D signaling in the brain and to develop more effective therapeutic strategies. Overall, maintaining appropriate vitamin D levels may represent a promising approach for supporting neurological health and potentially reducing the burden of neurodegenerative diseases.

## Figures and Tables

**Table 2:** Recent clinical studies on vitamin D in neurodegenerative and neurological disorders.

Design of the Study	Vitamin D Intervention	Clinical Outcomes	Reference
Parallel, Double-blind (DB), Randomized (R), Placebo-controlled (PC); *n* = 316 Clinically Isolated Syndrome (CIS) patients	Cholecalciferol 100,000 IU every 2 weeks (24 months)	Significant reduction in disease activity (60.3% vs. 74.1%); delayed MS conversion; prolonged relapse-free survival; reduced MRI lesion burden; HR 0.66; no increase in adverse events	[69]
DB, R, PC; *n* = 199 high-risk CIS patients	Vitamin D_3_ 1000–10,000 IU/day	No significant reduction in MS conversion; no improvement in relapse or MRI outcomes; safe and well tolerated	[70]
Randomized Controlled Trial (RCT); *n* = 46 PD patients	Vitamin D + probiotics (12 weeks)	Reduced pro-inflammatory cytokines and oxidative stress marker; increased IL-10 and total antioxidant capacity (TAC); improved Unified Parkinson’s Disease Rating Scale (UPDRS) scores, anxiety symptoms, and gastrointestinal function	[71]
DB, R, PC; *n* = 30 vitamin D-deficient PD patients	Vitamin D_3_ (~5000 IU every 5 days, 3 months)	Increased serum 25(OH)D levels; reduced Th17 cells and increased regulatory T cells; significant improvement in motor function (UPDRS, UPDRS-III)	[72]
Prospective cohort; *n* = 269, 229	Serum vitamin D status + supplementation	Vitamin D deficiency is associated with increased AD, vascular dementia (VD), and dementia risk; supplementation is associated with reduced incidence	[73]
Observational cohort; *n* = 364 PD patients	Serum 25(OH)D levels	U-shaped mortality relationship; optimal survival at ~75–100 nmol/L; both deficiency and excess increase mortality risk	[74]
Case-cohort; *n* ≈ 4000	Plasma 25(OH)D quartiles	No protective effect: the highest vitamin D quartile is associated with increased disabling dementia risk in the elderly subgroup	[75]
Prospective cohort; *n* = 366,160	Serum 25(OH)D levels	Higher vitamin D levels are associated with reduced AD, VD, and all-cause dementia risk; the is effect modified by sleep characteristics	[76]
DB, R, PC; *n* ≈ 2495 older adults	Vitamin D_3_ (1600–3200 IU/day, long-term)	No reduction in dementia incidence; no significant improvement in cognitive decline vs. placebo	[77]
Single-blind, R, PC; *n* = 159 ischemic stroke patients	Vitamin D3 2000 IU/day (6 weeks)	Increased serum 25(OH)D levels; improved functional recovery as indicated by higher Barthel Index scores during rehabilitation	[78]
Prospective cohort + Mendelian randomization	Serum 25(OH)D levels	Higher vitamin D associated with reduced stroke risk; MR supports causal relationship	[77]
Clinical observational; *n* = 160 stroke patients	Serum vitamin D levels	Lower vitamin D associated with worse MoCA and NIHSS scores; linked to post-stroke cognitive impairment	[79]
Propensity-matched cohort; *n* > 8000	Vitamin D deficiency status	Vitamin D deficiency is associated with increased risk of acute kidney injury, mortality, ICU admission, pneumonia, and progression to end-stage renal disease (ESRD) after stroke	[80]
Clinical study; *n* = 16 MS patients	Vitamin D supplementation	Reduced pro-inflammatory cytokine levels and improved immune balance; associated with decreased relapse-related inflammatory activity	[81]
Clinical trial; *n* = 35 MS patients	Vitamin D_3_ 50,000 IU/week	Reduced oxidative stress markers (MDA, TOS) and increased antioxidant enzymes (SOD, CAT, GPx)	[82]
Prospective cohort; *n* = 120 MS patients	Serum vitamin D levels	Low vitamin D (<20 ng/mL) is associated with higher relapse frequency	[83]
Observational study	Serum vitamin D levels	Each 10 nmol/L increase in vitamin D reduced relapse risk by ~6.7%	[84]
Observational; *n* = 132 Relapsing-Remitting Multiple Sclerosis (RRMS) patients	Vitamin D supplementation	Reduced brain atrophy; no significant effect on Expanded Disability Status Scale (EDSS), relapse rate, or MRI lesion activity	[85]

## Data Availability

No new data were created or analyzed in this study.
